# Bis(1-amino-4-methyl­pyridinium) bis­(1,2-dicyano­ethene-1,2-dithiol­ato-κ^2^ *S*,*S*′)nickelate(II)

**DOI:** 10.1107/S1600536808018886

**Published:** 2008-06-28

**Authors:** Jian-Lan Liu, Bing-Qian Yao, Shao-Ming Zhang

**Affiliations:** a Department of Applied Chemistry, College of Science, Nanjing University of Technology, Nanjing 210009, People’s Republic of China

## Abstract

The asymmetric unit of the title compound, (C_6_H_9_N_2_)_2_[Ni(C_4_N_2_S_2_)_2_], contains one half of an [Ni(mnt)_2_]^2−^ anion (mnt is maleonitrile­dithiol­ate or 1,2-dicyano­ethene-1,2-dithiol­ate) and one 1-amino-4-methyl­pyridinium cation. The Ni^II^ atom is located on an inversion centre. In the crystal structure, inter­molecular N—H⋯N hydrogen bonds link the mol­ecules.

## Related literature

For general background, see: Cassoux *et al.* (1991 ▶). For bond-length data, see: Allen *et al.* (1987 ▶).
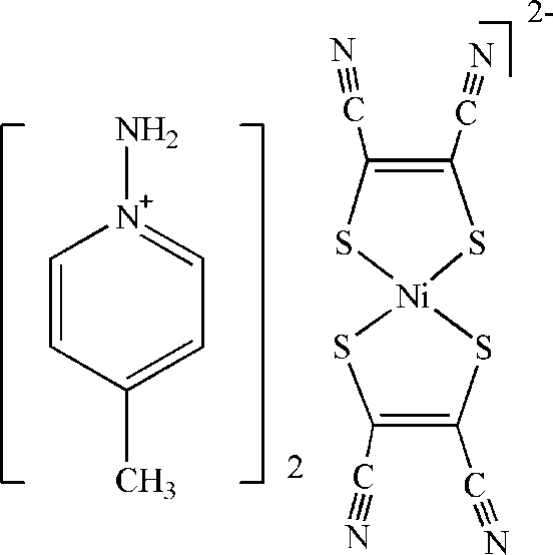


## Experimental

### 

#### Crystal data

(C_6_H_9_N_2_)_2_[Ni(C_4_N_2_S_2_)_2_]
*M* *_r_* = 557.39
Triclinic, 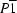

*a* = 7.678 (5) Å
*b* = 9.095 (6) Å
*c* = 9.665 (6) Å
α = 93.116 (7)°
β = 104.519 (8)°
γ = 108.813 (7)°
*V* = 611.7 (7) Å^3^
*Z* = 1
Mo *K*α radiation
μ = 1.16 mm^−1^
*T* = 296 (2) K
0.30 × 0.20 × 0.10 mm

#### Data collection

Bruker SMART CCD area-detector diffractometer
Absorption correction: multi-scan (*SADABS*; Bruker, 2000 ▶) *T*_min_ = 0.732, *T*_max_ = 0.892
3040 measured reflections
2097 independent reflections
1937 reflections with *I* > 2σ(*I*)
*R*_int_ = 0.137

#### Refinement

*R*[*F* ^2^ > 2σ(*F* ^2^)] = 0.065
*wR*(*F* ^2^) = 0.173
*S* = 1.04
2097 reflections
152 parameters
H-atom parameters constrained
Δρ_max_ = 0.82 e Å^−3^
Δρ_min_ = −1.48 e Å^−3^

### 

Data collection: *SMART* (Bruker, 2000 ▶); cell refinement: *SAINT* (Bruker, 2000 ▶); data reduction: *SAINT*=; program(s) used to solve structure: *SHELXS97* (Sheldrick, 2008 ▶); program(s) used to refine structure: *SHELXL97* (Sheldrick, 2008 ▶); molecular graphics: *ORTEP-3 for Windows* (Farrugia, 1997 ▶); software used to prepare material for publication: *SHELXTL* (Sheldrick, 2008 ▶).

## Supplementary Material

Crystal structure: contains datablocks global, I. DOI: 10.1107/S1600536808018886/hk2475sup1.cif

Structure factors: contains datablocks I. DOI: 10.1107/S1600536808018886/hk2475Isup2.hkl

Additional supplementary materials:  crystallographic information; 3D view; checkCIF report

## Figures and Tables

**Table 1:** Hydrogen-bond geometry (Å, °)

*D*—H⋯*A*	*D*—H	H⋯*A*	*D*⋯*A*	*D*—H⋯*A*
N4—H4*A*⋯N1^i^	0.86	2.35	3.151 (6)	155
N4—H4*B*⋯N2	0.86	2.58	3.075 (5)	118

Symmetry code: (i) 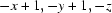.

**Fig. 1.**
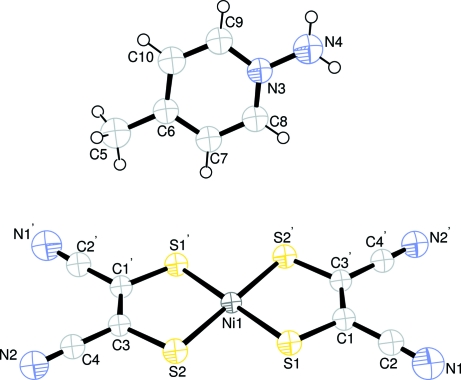
The molecular structure of the title molecule, with the atom-numbering scheme [symmetry code: (') -x, -y, -z].

**Fig. 2.**
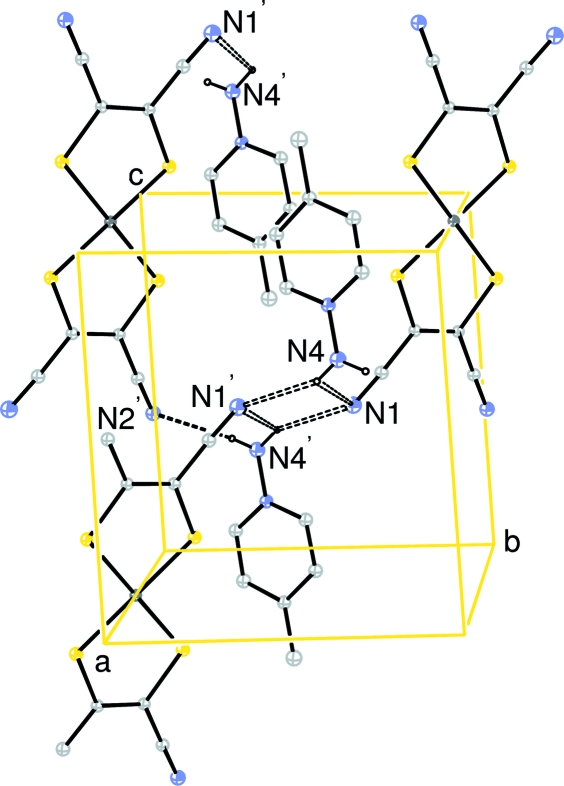
A partial packing diagram of (I). Hydrogen bonds are shown as dashed lines [symmetry code: (') -x, -y, -z]. H atoms not involved in hydrogen bonding have been omitted for clarity.

## supplementary crystallographic information

### Comment

Square-planar *M*[dithiolene]_2_ complexes have attracted extensive interests in the areas of conducting and magnetic materials, dyes, non-linear optics and catalysis (Cassoux *et al.*,  1991). We report herein the crystal structure of the title compound, (I).

The asymmetric unit of (I) (Fig. 1) contains one-half [Ni(mnt)_2_]^2-^ anion (where mnt is maleonitriledithiolate) and one 1-amino-4-methyl- pyridinium cation. The Ni^II^ atom is located at the inversion centre. The bond lengths (Allen *et al.*,  1987) and angles are within normal ranges.

In the crystal structure, intra- and intermolecular N-H···N hydrogen bonds (Table 2) link the molecules (Fig. 2), in which they may be effective in the stabilization of the structure.

### Experimental

For the preparation of the title compound, disodium maleonitriledithiolate (456 mg, 2.5 mmol) and nickel chloride hexahydrate (297 mg, 1.25 mmol) were mixed with water (20 ml) by stirring at room temperature. Subsequently, a solution of 1-amino-4-methylpyridinium iodide (590 mg, 2.5 mmol) in water (10 ml) was added to the mixture, and the red precipitate immediately formed was filtered off, and washed with water. The crude product was recrystallized in acetone (20 ml) to give red crystals. Crystals suitable for X-ray analysis were obtained by diffusing diethyl ether into the solution of (I) in acetone for 6 d. Anal. Calcd.: C, 43.10; H, 3.26; N, 20.10%. Found: C, 43.15; H, 3.29; N, 20.16%. FTIR data (KBr pellets, cm^-1^): 3025, 2928, 2920, 2199, 1577, 1490, 1399, 1125.

### Refinement

H atoms were positioned geometrically, with N-H = 0.86 Å (for NH_2_) and C-H= 0.93 and 0.96 Å for aromatic and methyl H, and constrained to ride on their parent atoms, with U_iso_(H) = xU_eq_(C,N), where x = 1.5 for methyl H, and x = 1.2 for all other H atoms.

### Crystal data

**Table:**

(C_6_H_9_N_2_)_2_[Ni(C_4_N_2_S_2_)_2_]	*Z* = 1
*M**_r_* = 557.39	*F*_000_ = 286
Triclinic, *P*1	*D*_x_ = 1.513 Mg m^−^^3^
Hall symbol: -P 1	Mo *K*α radiation λ = 0.71073 Å
*a* = 7.678 (5) Å	Cell parameters from 2479 reflections
*b* = 9.095 (6) Å	θ = 2.3–22.2º
*c* = 9.665 (6) Å	µ = 1.16 mm^−^^1^
α = 93.116 (7)º	*T* = 296 (2) K
β = 104.519 (8)º	Block, red
γ = 108.813 (7)º	0.30 × 0.20 × 0.10 mm
*V* = 611.7 (7) Å^3^	

### Data collection

**Table:**

Bruker SMART CCD area-detector diffractometer	2097 independent reflections
Radiation source: sealed tube	1937 reflections with *I* > 2σ(*I*)
Monochromator: graphite	*R*_int_ = 0.137
*T* = 296(2) K	θ_max_ = 25.0º
φ and ω scans	θ_min_ = 2.2º
Absorption correction: multi-scan(SADABS; Bruker, 2000)	*h* = −8→9
*T*_min_ = 0.732, *T*_max_ = 0.892	*k* = −10→10
3040 measured reflections	*l* = −11→8

### Refinement

**Table:**

Refinement on *F*^2^	Secondary atom site location: difference Fourier map
Least-squares matrix: full	Hydrogen site location: inferred from neighbouring sites
*R*[*F*^2^ > 2σ(*F*^2^)] = 0.065	H-atom parameters constrained
*wR*(*F*^2^) = 0.173	*w* = 1/[σ^2^(*F*_o_^2^) + (0.1404*P*)^2^] where *P* = (*F*_o_^2^ + 2*F*_c_^2^)/3
*S* = 1.04	(Δ/σ)_max_ < 0.001
2097 reflections	Δρ_max_ = 0.82 e Å^−^^3^
152 parameters	Δρ_min_ = −1.48 e Å^−^^3^
Primary atom site location: structure-invariant direct methods	Extinction correction: none

### Special details

**Table:**

Geometry. All e.s.d.'s (except the e.s.d. in the dihedral angle between two l.s. planes) are estimated using the full covariance matrix. The cell e.s.d.'s are taken into account individually in the estimation of e.s.d.'s in distances, angles and torsion angles; correlations between e.s.d.'s in cell parameters are only used when they are defined by crystal symmetry. An approximate (isotropic) treatment of cell e.s.d.'s is used for estimating e.s.d.'s involving l.s. planes.
Refinement. Refinement of F^2^ against ALL reflections. The weighted R-factor wR and goodness of fit S are based on F^2^, conventional R-factors R are based on F, with F set to zero for negative F^2^. The threshold expression of F^2^ > 2sigma(F^2^) is used only for calculating R-factors(gt) etc. and is not relevant to the choice of reflections for refinement. R-factors based on F^2^ are statistically about twice as large as those based on F, and R- factors based on ALL data will be even larger.

### Fractional atomic coordinates and isotropic or equivalent isotropic displacement parameters (Å^2^)

**Table:**

	*x*	*y*	*z*	*U*_iso_*/*U*_eq_	
Ni1	0.5000	1.0000	0.0000	0.0326 (2)	
S1	0.53316 (12)	0.82630 (9)	−0.14377 (8)	0.0430 (3)	
S2	0.62570 (12)	0.89841 (9)	0.18019 (8)	0.0441 (3)	
N1	0.3775 (6)	0.6494 (4)	−0.5237 (4)	0.0726 (10)	
N2	0.8189 (5)	0.9732 (4)	0.5790 (3)	0.0570 (7)	
N3	0.8790 (4)	0.6656 (3)	0.8401 (3)	0.0463 (6)	
N4	0.8879 (5)	0.6838 (4)	0.6984 (4)	0.0633 (8)	
H4A	0.8169	0.6097	0.6282	0.076*	
H4B	0.9644	0.7692	0.6817	0.076*	
C1	0.4152 (4)	0.8525 (4)	−0.3142 (3)	0.0390 (6)	
C2	0.3924 (5)	0.7418 (4)	−0.4332 (3)	0.0476 (7)	
C3	0.6515 (4)	1.0259 (3)	0.3304 (3)	0.0373 (6)	
C4	0.7452 (4)	0.9981 (4)	0.4683 (3)	0.0433 (7)	
C5	0.8723 (7)	0.6146 (5)	1.2737 (4)	0.0694 (11)	
H5A	0.9882	0.6007	1.3268	0.104*	
H5B	0.7639	0.5245	1.2731	0.104*	
H5C	0.8615	0.7068	1.3187	0.104*	
C6	0.8775 (5)	0.6333 (4)	1.1208 (4)	0.0488 (7)	
C7	0.7604 (5)	0.5155 (4)	1.0066 (4)	0.0508 (7)	
H7	0.6800	0.4223	1.0251	0.061*	
C8	0.7615 (5)	0.5345 (4)	0.8667 (4)	0.0511 (8)	
H8	0.6802	0.4556	0.7909	0.061*	
C9	0.9972 (5)	0.7824 (4)	0.9466 (4)	0.0516 (8)	
H9	1.0795	0.8726	0.9252	0.062*	
C10	0.9965 (5)	0.7687 (4)	1.0869 (4)	0.0564 (9)	
H10	1.0764	0.8509	1.1601	0.068*	

### Atomic displacement parameters (Å^2^)

**Table:**

	*U*^11^	*U*^22^	*U*^33^	*U*^12^	*U*^13^	*U*^23^
Ni1	0.0304 (3)	0.0274 (3)	0.0316 (3)	0.0051 (2)	0.0022 (2)	−0.0029 (2)
S1	0.0501 (5)	0.0389 (4)	0.0369 (4)	0.0191 (3)	0.0041 (3)	−0.0026 (3)
S2	0.0558 (5)	0.0376 (4)	0.0348 (4)	0.0194 (4)	0.0031 (3)	−0.0010 (3)
N1	0.091 (3)	0.058 (2)	0.0556 (18)	0.0214 (18)	0.0098 (17)	−0.0172 (15)
N2	0.0604 (18)	0.0588 (18)	0.0416 (15)	0.0165 (14)	0.0021 (12)	0.0067 (12)
N3	0.0428 (14)	0.0357 (13)	0.0576 (15)	0.0130 (11)	0.0119 (11)	−0.0003 (11)
N4	0.078 (2)	0.0500 (17)	0.0605 (18)	0.0180 (16)	0.0238 (16)	0.0052 (13)
C1	0.0360 (14)	0.0370 (15)	0.0354 (14)	0.0068 (11)	0.0052 (11)	−0.0055 (11)
C2	0.0506 (18)	0.0385 (16)	0.0421 (16)	0.0100 (13)	0.0029 (13)	−0.0063 (12)
C3	0.0334 (14)	0.0344 (14)	0.0342 (13)	0.0058 (11)	0.0020 (10)	−0.0028 (10)
C4	0.0400 (16)	0.0402 (15)	0.0404 (15)	0.0073 (12)	0.0051 (12)	0.0007 (12)
C5	0.075 (3)	0.064 (3)	0.063 (2)	0.027 (2)	0.0076 (19)	0.0050 (18)
C6	0.0418 (17)	0.0439 (16)	0.0552 (18)	0.0176 (13)	0.0028 (13)	−0.0011 (13)
C7	0.0487 (18)	0.0321 (15)	0.0605 (19)	0.0042 (13)	0.0111 (14)	−0.0011 (13)
C8	0.0461 (18)	0.0336 (15)	0.0620 (19)	0.0060 (13)	0.0085 (14)	−0.0080 (13)
C9	0.0404 (17)	0.0317 (15)	0.071 (2)	0.0030 (12)	0.0104 (15)	−0.0006 (14)
C10	0.0464 (18)	0.0393 (17)	0.063 (2)	0.0053 (14)	−0.0033 (15)	−0.0110 (14)

### Geometric parameters (Å, °)

**Table:**

Ni1—S1	2.1655 (12)	C5—H5C	0.9600
Ni1—S1^i^	2.1655 (12)	C6—C10	1.388 (5)
Ni1—S2	2.1738 (11)	C6—C7	1.388 (5)
Ni1—S2^i^	2.1738 (11)	C7—C8	1.375 (5)
S1—C1	1.739 (3)	C7—H7	0.9300
S2—C3	1.737 (3)	C8—N3	1.326 (4)
C1—C3^i^	1.361 (4)	C8—H8	0.9300
C1—C2	1.423 (4)	C9—N3	1.343 (4)
C2—N1	1.137 (5)	C9—C10	1.369 (5)
C3—C1^i^	1.361 (4)	C9—H9	0.9300
C3—C4	1.426 (4)	C10—H10	0.9300
C4—N2	1.149 (4)	N3—N4	1.404 (4)
C5—C6	1.506 (5)	N4—H4A	0.8600
C5—H5A	0.9600	N4—H4B	0.8600
C5—H5B	0.9600		
			
S1—Ni1—S1^i^	180.00 (3)	C10—C6—C7	117.0 (3)
S1—Ni1—S2	88.08 (5)	C10—C6—C5	122.0 (3)
S1^i^—Ni1—S2	91.92 (5)	C7—C6—C5	121.0 (4)
S1—Ni1—S2^i^	91.92 (5)	C8—C7—C6	120.9 (3)
S1^i^—Ni1—S2^i^	88.08 (5)	C8—C7—H7	119.5
S2—Ni1—S2^i^	180.0	C6—C7—H7	119.5
C1—S1—Ni1	103.30 (12)	N3—C8—C7	119.8 (3)
C3—S2—Ni1	103.34 (12)	N3—C8—H8	120.1
C3^i^—C1—C2	122.5 (3)	C7—C8—H8	120.1
C3^i^—C1—S1	120.5 (2)	N3—C9—C10	120.0 (3)
C2—C1—S1	117.0 (3)	N3—C9—H9	120.0
N1—C2—C1	176.8 (4)	C10—C9—H9	120.0
C1^i^—C3—C4	122.4 (3)	C9—C10—C6	120.6 (3)
C1^i^—C3—S2	120.3 (2)	C9—C10—H10	119.7
C4—C3—S2	117.4 (2)	C6—C10—H10	119.7
N2—C4—C3	178.9 (4)	C8—N3—C9	121.7 (3)
C6—C5—H5A	109.5	C8—N3—N4	120.4 (3)
C6—C5—H5B	109.5	C9—N3—N4	117.8 (3)
H5A—C5—H5B	109.5	N3—N4—H4A	120.0
C6—C5—H5C	109.5	N3—N4—H4B	120.0
H5A—C5—H5C	109.5	H4A—N4—H4B	120.0
H5B—C5—H5C	109.5		

Symmetry codes: (i) −*x*+1, −*y*+2, −*z*.

### Hydrogen-bond geometry (Å, °)

**Table:**

*D*—H···*A*	*D*—H	H···*A*	*D*···*A*	*D*—H···*A*
N4—H4A···N1^ii^	0.86	2.35	3.151 (6)	155
N4—H4B···N2	0.86	2.58	3.075 (5)	118

Symmetry codes: (ii) −*x*+1, −*y*+1, −*z*.
